# CINSARC and Sarculator in Patients with Primary Retroperitoneal Sarcoma: A Combined Analysis of Single-Institution Data and the EORTC-STBSG-62092 Trial (STRASS)

**DOI:** 10.1158/1078-0432.CCR-25-0099

**Published:** 2025-05-27

**Authors:** Dario Callegaro, Gabriele Tinè, Felix Boakye Oppong, Axelle Nzokirantevye, Saskia Litière, Stefano Percio, Andrea Carenzo, Loris De Cecco, Frederic Chibon, Silvia Brich, Alessia Bertolotti, Paola Collini, Anna Maria Frezza, Paul Huang, Rick Haas, Sylvie Bonvalot, Winan J. van Houdt, Rosalba Miceli, Sandro Pasquali, Alessandro Gronchi

**Affiliations:** 1 Department of Surgery, Fondazione IRCCS Istituto Nazionale dei Tumori, Milan, Italy.; 2 Department of Data Science and Epidemiology, Fondazione IRCCS Istituto Nazionale dei Tumori, Milan, Italy.; 3 The European Organisation for Research and Treatment of Cancer (EORTC) Headquarters, Brussels, Belgium.; 4 Molecular Pharmacology, Department of Experimental Oncology, Fondazione IRCCS Istituto Nazionale dei Tumori, Milan, Italy.; 5 Integrated Biology of Rare Tumors Unit, Department of Experimental Oncology, Fondazione IRCCS Istituto Nazionale dei Tumori, Milan, Italy.; 6 Oncogenesis of Sarcomas, INSERM UMR1037, Cancer Research Centre of Toulouse, Toulouse, France.; 7 Soft Tissue Tumor Pathology Unit, Department of Advanced Diagnostics, Fondazione IRCCS Istituto Nazionale dei Tumori, Milan, Italy.; 8 Department of Medical Oncology, Fondazione IRCCS Istituto Nazionale dei Tumori, Milan, Italy.; 9 Division of Molecular Pathology, The Institute of Cancer Research, London, United Kingdom.; 10 Department of Radiotherapy, The Netherlands Cancer Institute, Amsterdam, the Netherlands.; 11 Department of Radiotherapy, The Leiden University Medical Center, Leiden, the Netherlands.; 12 Department of Surgical Oncology, Institut Curie, Paris, France.; 13 Department of Surgery, The Netherlands Cancer Institute, Amsterdam, the Netherlands.

## Abstract

**Purpose:**

The Complexity INdex in SARComas (CINSARC) predicts the metastatic risk in patients with soft-tissue sarcoma. The aims of this study were to provide the first independent validation of CINSARC in patients with retroperitoneal sarcoma (RPS) and evaluate whether CINSARC could enhance the performance of Sarculator.

**Experimental Design:**

A retrospective cohort included patients with primary localized RPS resected with curative intent (2011–2015) at a single institution. The STRASS cohort comprised patients from the surgery-only arm of the EORTC-STBSG-62092 (STRASS) trial who had undergone CINSARC categorization. Patients were classified as CINSARC low-risk (C1) versus high-risk (C2). Primary study endpoints were overall survival (OS) and disease-free survival (DFS). Sarculator performance was assessed in terms of discrimination (the Harrell C-index) and calibration (calibration plots and the Brier score) before and after adding CINSARC.

**Results:**

The study cohorts included 104 and 69 patients, respectively, with similar OS. In a pooled cohort, in multivariable analysis for OS considering Sarculator and CINSARC, only Sarculator was significantly associated with OS [HR, 1.93; 95% confidence interval (CI), 1.35–2.74; *P* < 0.001]. In multivariable analysis for DFS, both Sarculator (HR, 1.51; 95% CI, 1.09–2.09; *P* = 0.013) and CINSARC (HR, 2.01; 95% CI, 1.26–3.23; *P* = 0.004) were significantly associated with DFS. However, the addition of CINSARC did not improve Sarculator’s discrimination or calibration for either OS or DFS.

**Conclusions:**

This study validates CINSARC as a prognostic predictor for OS and DFS in patients with primary RPS. CINSARC did not improve the performance of Sarculator, suggesting that its addition to the Sarculator may not provide added clinical benefit.

**Translational Relevance**

This study utilized a retrospective single-institution cohort of patients with primary localized retroperitoneal sarcomas (RPS) and the surgery-only arm of the EORTC-STBSG-62092 (STRASS) trial to evaluate the prognostic relevance of the Complexity INdex in SARComas (CINSARC) and its potential to enhance the prognostic accuracy of the Sarculator nomogram. CINSARC, a transcriptomic signature that independently predicts metastatic risk in patients with soft-tissue sarcoma, has not been previously validated in RPS, and its added prognostic value to Sarculator remains uncertain. Our findings demonstrate that CINSARC is a significant prognostic marker for overall and disease-free survival in patients with primary RPS undergoing surgery. However, its integration into the Sarculator did not improve the model’s discrimination or calibration, indicating that CINSARC may not provide additional clinical utility when combined with the Sarculator in this patient population.

## Introduction

The prognostication of patients with retroperitoneal sarcoma (RPS) is primarily based on clinical factors such as tumor characteristics (size, grade, histology, and multifocality), patient age, and the quality of surgical treatment (1–3). These variables have been integrated into nomograms that provide individualized patient prognoses after resection (4–10). One such tool, the Sarculator, predicts 7-year overall survival (OS) and disease-free survival (DFS) following surgery for primary RPS. Sarculator has been validated in multiple independent series, demonstrating reliability and generalizability (4, 11, 12). Recent evidence suggests that the performance of these clinically based nomograms may be enhanced by incorporating biomarkers with meaningful prognostic significance (13). The Complexity INdex in SARComas (CINSARC) is a transcriptomic signature developed by Chibon and colleagues (14) in 2010 to predict outcomes in patients with soft-tissue sarcoma (STS). Based on the expression of 67 genes involved in chromosome biogenesis, mitosis regulation, and chromosome segregation, CINSARC classifies patients into two groups: C1 (low CINSARC score, favorable prognosis) and C2 (high CINSARC score, poor prognosis; ref. 15). This signature has been shown to independently predict metastatic risk in STS, even when adjusted for Fédération Nationale des Centres de Lutte Contre le Cancer (FNCLCC) grade (14, 16), as well as in gastrointestinal stromal tumor (17), synovial sarcoma (18), rhabdomyosarcoma (19), and leiomyosarcoma (LMS; ref. 18, 20). However, CINSARC has not yet been specifically validated in patients with RPS, and interactions with Sarculator remains to be elucidated (21). The aims of the present study were to provide the first independent validation of CINSARC in two cohorts of patients with primary RPS and evaluate whether the addition of CINSARC to the Sarculator nomograms could improve their performance.

## Materials and Methods

### Patients

The Fondazione IRCCS Istituto Nazionale dei Tumori (INT), Milan, Italy, cohort included all patients with primary (nonrecurrent and nonmetastatic) localized RPS, resected between 2011 and 2015 and enrolled in the retrospective cohort of the SARCOMICS study. SARCOMICS, initiated in 2018 at INT, is an observational study aimed at investigating whether the integration of radiomic, genomic, and immunologic markers could enhance the performance of clinical-based nomograms. The study consists of both retrospective and prospective cohorts of patients with primary RPS or primary extremity/superficial trunk STS treated with curative-intent surgery. The retrospective cohort included patients with primary (nonrecurrent and nonmetastatic) localized RPS, resected between 2011 and 2015, with formalin-fixed paraffin-embedded (FFPE) material of the surgical specimen available in the institutional biobank and preoperative CT-scan DICOM files available for radiomic analysis. Patients with Ewing sarcoma family tumors, pediatric rhabdomyosarcomas, desmoid tumors, gynecologic sarcomas, and gastrointestinal stromal tumors were excluded. Clinical data were collected from a prospectively maintained database with more than 10,000 records. Adjuvant/neoadjuvant chemotherapy and/or radiotherapy were administered based on multidisciplinary clinical decisions. Chemotherapy regimens were either standard at the time or part of ongoing institutional or multi-institutional clinical studies. Radiotherapy was delivered via an external beam at a median dose of 50 Gy in the preoperative setting. Postoperative follow-up consisted of a CT scan of the chest, abdomen, and pelvis every 4 months for the first 2 years, every 6 months from years 3 to 5, and annually thereafter.

The European Organisation for Research and Treatment of Cancer (EORTC; RRID: SCR_004070) Soft Tissue and Bone Sarcoma Group run a randomized trial that compared radiotherapy plus surgery versus surgery alone in RPS. The EORTC-STBSG-62092 (STRASS) cohort included patients enrolled in the surgery-only arm of the EORTC-STBSG-62092 (STRASS) trial and who had undergone CINSARC categorization (22, 23). The phase III STRASS trial has been described elsewhere (22). Briefly, this study randomized 266 patients with primary localized RPS to receive either surgery alone (standard arm) or preoperative radiotherapy followed by surgery (experimental arm). STRASS was conducted across 13 countries in Europe and North America, enrolling 266 patients between 2012 and 2017. At a median follow-up of 43 months, no significant difference in abdominal recurrence-free survival was observed between the two arms. *Post hoc* subgroup analyses and the analysis of combined in-trial and outside-trial patients suggested possible benefits of radiotherapy for patients with G1 and G2 liposarcoma (22, 23). In the STRASS cohort, chemotherapy was not allowed. Radiotherapy was administered at a dose of 50.4 Gy in 28 once-daily 1.8-Gy fractions. Postsurgery follow-up included CT scans every 3 months during the first year and every 6 months thereafter, until recurrence or death.

The FNCLCC grading system was applied. Histologic types were grouped into well-differentiated liposarcoma (WDLPS), dedifferentiated liposarcoma (DDLPS), LMS, solitary fibrous tumor (SFT), malignant peripheral-nerve sheath tumor, undifferentiated pleomorphic sarcoma, and other sarcomas.

The study has been approved by the Institutional Ethics Committee at Fondazione IRCCS Istituto Nazionale Tumori, Milan, Italy (ID: INT 77/18). Procedures performed were in accordance with the ethical standards of the institutional and/or national research committee and with the 1964 Helsinki Declaration and its later amendments. Informed consent was obtained from all prospective participants, including patients in the EORTC-STBSG-62092 (STRASS) trial. For retrospective cases in which obtaining consent was not feasible (e.g., due to patient death), the study was conducted in accordance with current legislation, which permits the use of such data for research purposes.

### Procedures

In the INT cohort, patients were classified into the two CINSARC categories (C1 and C2) following the CINSARC classifier (24), coded in the hacksig R package (RRID: SCR_025833; ref. 25).

In the INT cohort, each surgical specimen was reviewed by a pathologist with expertise in STS, and a representative area from the tumor and normal tissue was selected. A hematoxylin and eosin control slide was prepared to assess the quality of the material. In the STRASS cohort, pathology review was centralized. In both cohorts, for DDLPS, the dedifferentiated component was used to reflect the aggressiveness of the disease. In the INT cohort, RNA was extracted from FFPE material using miRNeasy FFPE Kit (Qiagen) and quality-checked and quantified using the TapeStation 4150 (Agilent) and Qubit (Thermo Fisher Scientific), respectively. Total RNA was profiled for gene expression by bulk RNA sequencing analysis. RNA libraries for sequencing were generated using the QuantSeq 3′ mRNA-Seq Library Prep Kit (Lexogen), following the manufacturer’s instructions. Initially, RNA samples were reverse-transcribed using an oligo-dT primer with Illumina-compatible indexes. After second-strand synthesis using random primers that incorporated Illumina-compatible linker sequences and purification with magnetic beads, the libraries underwent amplification to incorporate the full adapter sequences required for cluster generation. The libraries were then purified again and equimolarly pooled. Sequencing was performed on a NextSeq 500 (Illumina) to generate single-end reads, and the resulting FASTQ files were processed using the BlueBee Genomics Platform (Lexogen).

STRASS samples were processed as described previously (26).

### Sarculator

The Sarculator nomograms for primary RPS were applied to all patients to calculate OS and DFS scores based on the nomogram-specific clinical variables (patient age, tumor size, FNCLCC grade, histology, multifocality, and completeness of resection for OS and tumor size, FNCLCC grade, histology, and multifocality for DFS; ref. 27).

### Statistical methods

The primary clinical endpoints of our study were OS and DFS. OS was defined as the time interval between surgery and death from any cause, with censoring at the date of last follow-up for alive patients. DFS was defined as the time interval between surgery and local or distant recurrence or death from any cause, with censoring at the date of last follow-up for patients alive without any recurrence. Survival curves were estimated with the Kaplan–Meier method and statistically compared with the log-rank test. The Cox regression model was used for multivariable analyses, and the results are reported in terms of HR, corresponding 95% confidence interval (CI), and *P* value for the Wald test.

The performance of the CINSARC was tested on the study cohort. To assess whether CINSARC could enhance the performance of Sarculator, we used a multivariable Cox model that included both Sarculator and CINSARC, comparing the results with those from a model including only Sarculator. Calibration was assessed with calibration plots, which are graphical representations of the correlation between predicted and observed outcomes. In a perfectly calibrated model, calibration plots would insist on the 45-degree line. If the plots deviate from the 45-degree line, the model is either too optimistic or pessimistic. Nomogram’s overall accuracy was estimated by the Brier Score (BS), which is equal to the sum of two components: one correspondent to calibration and the other to the discriminative ability, related to the area under the ROC curve (AUC; ref. 28). BS ranges from 0 to 1: a lower BS indicates a better model accuracy in terms of both calibration and discriminative ability. The latter was also assessed using the most known Harrell C-index together with its 95% bootstrap CI. The Harrell C-index spans from 1 (perfect discrimination) for a model that is always able, given a random couple of patients, to assign the worst prognosis to the patient who will experience the event first to 0.5 for a model in which the ability to identify the patient with worse prognosis is not better than chance. To further evaluate models’ performance, the 5- and 10-year time-dependent AUC were also calculated. The C-index provides information about the overall discriminative ability of a survival model; conversely, the time-dependent AUC assesses the discriminative ability at a specific time point of interest. The time-dependent AUC for each interval reflects the model’s predictive accuracy within that particular time window, representing an additional metric that allows for a comprehensive evaluation of the model’s predictive ability. Furthermore, the bootstrapped delta BS between nomogram alone and nomogram with CINSARC was computed to assess whether CINSARC improves calibration.

To increase the power of our analysis, the INT and STRASS datasets were pooled with an individual patient data approach. The Cox models on the pooled datasets were fitted both without and with inclusion of a random component to take into account the possible heterogeneity between INT and STRASS. In the pooled cohort, the prognostic effect of CINSARC or Sarculator was also assessed across histologies and tumor grades in subgroup analyses.

The statistical analyses were performed using the SAS (RRID: SCR_008567) and R software (RRID: SCR_001905; ref. 29). We considered a statistical test as significant when the corresponding *P* value was <5%.

### Data availability

Transcriptomic data of the INT dataset were deposited at Gene Expression Omnibus (accession number GSE296413). The STRASS dataset is available from the EORTC Soft Tissue and Bone Sarcoma Group (https://www.eortc.org/research_field/sarcoma/) on reasonable request through the EORTC website (https://www.eortc.be/services/forms/erp/request.aspx). Raw data, such as the data behind the figures, is available upon request to the corresponding author.

## Results

### Clinicopathologic features: INT and STRASS cohorts

Between 2011 and 2015, 235 patients with primary localized RPS were resected at INT. Of these, 117 (50%) patients had material suitable for the CINSARC analysis and were included in the SARCOMICS retrospective cohort. Thirteen patients who overlapped with the STRASS cohort were excluded, leaving 104 patients in the INT cohort. Based on their gene expression profile, 56 patients (54%) were classified as CINSARC low-risk (C1), whereas 48 (46%) were classified as CINSARC high-risk (C2).

Between 2012 and 2017, 133 patients were enrolled in the surgery-only arm of the STRASS trial. Of these, 69 (52%) had the FFPE material suitable for CINSARC determination and were included in the STRASS cohort of this study. According to CINSARC, 46 (67%) were classified as low-risk (C1) and 23 (33%) as high-risk (C2).

The INT and STRASS cohorts were comparable in terms of sex, age, histology, and tumor grade (Table 1). However, STRASS patients had smaller tumors, with a median tumor size of 16.7 cm compared with 22.5 cm in the INT cohort (*P* < 0.001). All resections in both cohorts were macroscopically complete (R0 or R1), and tumors were all unifocal.

**Table 1.** Baseline characteristics of the INT and STRASS cohorts.

Variable	STRASS, *N* = 69^a^	INT, *N* = 104^a^	*P* value^b^
Age	61.0 (50.0, 69.0)	64.0 (56.0, 71.3)	0.131
Sex			0.088
Male	39 (56.52%)	72 (69.23%)	
Female	30 (43.48%)	32 (30.77%)	
Histology			0.117
DDLPS	32 (46.38%)	47 (45.19%)	
WDLPS	17 (24.64%)	37 (35.58%)	
LMS	14 (20.29%)	13 (12.50%)	
MPNST	2 (2.90%)	2 (1.92%)	
Other^c^	3 (4.35%)	0 (0.00%)	
SFT	0 (0.00%)	3 (2.88%)	
UPS	1 (1.45%)	2 (1.92%)	
Tumor size (mm)	16.7 (12.4, 20.0)	22.5 (15.8, 30.0)	<0.001
Grading			0.422
1	23 (43.40%)	39 (37.50%)	
2	22 (41.51%)	40 (38.46%)	
3	8 (15.09%)	25 (24.04%)	
Missing	16	0	
Chemotherapy			0.006
Yes	0 (0.00%)	13 (12.5%)	
No	69 (100.00%)	91 (87.5%)	
Radiotherapy			0.003
Yes	0 (0.00%)	15 (14.4%)	
No	69 (100.00%)	89 (85.6%)	
Status at last FU			0.017
Alive	56 (81.16%)	67 (64.42%)	
Dead	13 (18.84%)	37 (35.58%)	
Local relapse/distant metastasis			0.058
Yes	31 (44.93%)	62 (59.62%)	
No	38 (55.07%)	42 (40.38%)	

Abbreviations: FU, follow-up; MPNST, malignant peripheral-nerve sheath tumor; UPS, undifferentiated pleomorphic sarcoma.

a Statistics: median (Q1, Q3); *n* (%).

b Wilcoxon rank-sum test, Fisher exact test; and Pearson *χ*^2^ test.

c Two myxoid liposarcoma and one liposarcoma not further specified.

### Survival analysis: INT and STRASS cohorts

The median follow-up was 108.0 months (IQR, 98.0–120.4) in the INT cohort and 47.4 months (IQR, 29.0–59.6) in the STRASS cohort. The 5-year OS was 75.8% (95% CI, 67.9–84.5; Supplementary Fig. S1A) in the INT cohort and 76.4% (95% CI, 65.1–89.7; Supplementary Fig. S1B) in the STRASS cohort.

When stratified by CINSARC, in the INT cohort, 5-year OS for C1 patients was 87.2% (95% CI, 79.4–95.9), whereas for C2 patients, it was 64.3% (95% CI, 52.6–78.6; Supplementary Fig. S1C). In the STRASS cohort, 5-year OS was 85.1% (95% CI, 74.6–97.1) for C1 patients and 56.5% (95% CI, 33.5–95.3) for C2 patients (Supplementary Fig. S1D).

In the INT cohort, 5-year DFS for C1 patients was 74.5% (95% CI, 54.4–86.1), whereas for C2 patients, it was 44.3% (95% CI, 32.9–59.8; Supplementary Fig. S2A). Similarly, in the STRASS cohort, 5-year DFS was 61.1% (95% CI, 45.9–81.4) for C1 patients and 8.3% (95% CI, 1.3–51.0.7) for C2 patients (Supplementary Fig. S2B).

### Clinicopathologic features: Pooled cohort

Given the similarity in baseline factors and OS between the INT and STRASS cohorts, the two were combined to form a pooled cohort of 173 patients. Baseline characteristics of the pooled cohort, stratified by CINSARC, are summarized in Table 2. Patients classified as C2 were generally older (*P* = 0.037), were more likely female (*P* < 0.001), had fewer cases of WDLPS and more cases of LMS (*P* < 0.001), had a higher proportion of high-grade tumors (*P* < 0.001), were more likely to have received chemotherapy (*P* = 0.008), and had lower predicted OS and DFS using Sarculator (*P* < 0.001; Supplementary Fig. S3). Distribution of LMS and DDLPS by grade according to the CINSARC risk group is detailed in Supplementary Table S1.

**Table 2.** Baseline characteristics of the pooled cohort stratified by CINSARC.

Variable	C1, *N* = 102^a^	C2, *N* = 71^a^	*P* value^b^
Age	62.5 (53.0, 68.0)	64.0 (56.0, 73.0)	0.037
Sex			<0.001
Male	62 (60.8%)	22 (39.1%)	
Female	40 (39.2%)	49 (69.0%)	
Histology			<0.001
DDLPS	47 (46.1%)	32 (45.1%)	
WDLPS	43 (42.2%)	11 (15.5%)	
LMS	5 (4.9%)	22 (31.0%)	
MPNST	2 (2.0%)	2 (2.8%)	
Other	2 (2.0%)	1 (1.4%)	
SFT	3 (2.9%)	0 (0%)	
UPS	0 (0.0%)	3 (4.2%)	
Tumor size (mm)	20.0 (15.0, 27.0)	19.0 (12.0, 27.0)	0.463
Grading			<0.001
1	48 (47.0%)	14 (19.7%)	
2	36 (35.3%)	26 (36.6%)	
3	6 (5.9%)	27 (38.0%)	
Missing	12	4	
Completeness of resection			
Complete	102 (100.0%)	71 (100.0%)	
Incomplete	0 (0.0%)	0 (0.0%)	
Chemotherapy			0.008
Yes	3 (2.9%)	10 (14.1%)	
No	99 (97.1%)	61 (85.9%)	
Radiotherapy			0.593
Yes	10 (9.8%)	5 (7.0%)	
No	92 (90.2%)	66 (93%)	
Status at last FU			0.004
Alive	81 (79.4%)	42 (59.2%)	
Dead	21 (20.6%)	29 (40.8%)	
Local relapse/distant metastasis			<0.001
Yes	43 (42.2%)	50 (70.4%)	
No	59 (47.8%)	21 (29.6%)	
5-year prOS			<0.001
Median (IQR)	0.9 (0.8, 0.9)	0.7 (0.5, 0.8)	
Missing^c^	12	4	
10-year prOS			<0.001
Median (IQR)	0.8 (0.6, 0.9)	0.5 (0.3, 0.7)	
Missing^c^	12	4	
5-year prDFS			<0.001
Median (IQR)	0.8 (0.6, 0.8)	0.5 (0.3, 0.6)	
Missing^c^	12	4	
10-year prDFS			<0.001
Median (IQR)	0.6 (0.4, 0.7)	0.3 (0.1, 0.5)	
Missing^c^	12	4	

Abbreviations: FU, follow-up; MPNST, malignant peripheral-nerve sheath tumor; prDFS, predicted DFS; prOS, predicted OS; UPS, undifferentiated pleomorphic sarcoma.

a Statistics: median (Q1, Q3); *n* (%).

b Wilcoxon rank-sum test, Fisher exact test, and Pearson *χ*^2^ test.

c Missing data for at least one nomogram variable.

### Survival analysis: Pooled cohort

**Figure 1.**
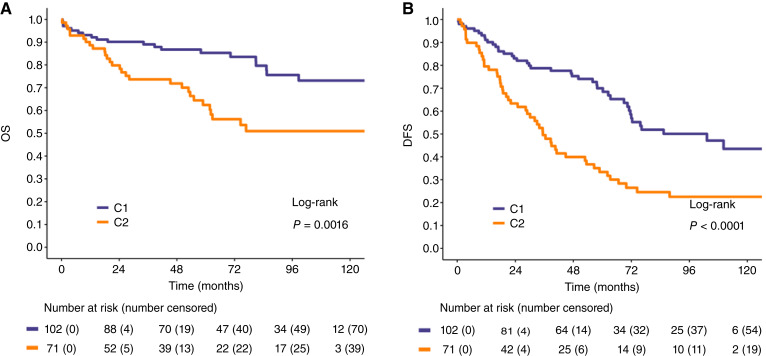
OS (**A**) and DFS (**B**) curves in the pooled cohort according to CINSARC.

The median follow-up of the pooled cohort was 85.1 months (IQR, 53.2–108.4). Five-year OS was 85.3% (78.4–92.8) in C1 patients and 62.4% (51.4–75.8) in C2 patients (*P* = 0.002; Fig. 1A). In the multivariable Cox model for OS, which included both Sarculator and CINSARC (Table 3), only Sarculator was significantly associated with OS (HR, 1.93; 95% CI, 1.35–2.74; *P* < 0.001). These results were consistent even when a random effect was introduced in the model accounting for cohort heterogeneity between INT and STRASS using a Cox model with random effects (Sarculator: HR, 1.93; 95% CI, 1.35–2.75; *P* < 0.001 and CINSARC: HR, 1.65; 95% CI, 0.84–3.24; *P* = 0.142).

**Table 3.** Results from the multivariable Cox models on the two endpoints.

Prognostic variables	OS			DFS		
	HR	95% CI	*P* value	HR	95% CI	*P* value
Sarculator nomogram score^a^	1.93	1.35–2.74	<0.001	1.51	1.09–2.09	0.013
CINSARC						
C1	Ref			Ref		
C2	1.65	0.84–3.23	0.141	2.01	1.26–3.23	0.004

a The HR for Sarculator reflects unitary increases in the nomogram score.

Five-year DFS was 70.0% (95% CI, 61.1–80.1) in C1 patients and 33.4% (95% CI, 23.6–47.2) in C2 patients (*P* < 0.001; Fig. 1B). In the multivariable Cox model for DFS (Table 3), both Sarculator (HR, 1.51; 95% CI, 1.09–2.09; *P* = 0.013) and CINSARC (HR, 2.01; 95% CI, 1.26–3.23; *P* = 0.004) were associated with DFS. Including cohorts as random effects in these models slightly increased the HR due to the relatively better DFS in the INT cohort and the stronger effect of CINSARC in the STRASS cohort (Sarculator: HR, 1.62; 95% CI, 1.16–2.25; *P* = 0.004 and CINSARC: HR, 2.24; 95% CI, 1.39–3.62; *P* = 0.001).

To explore the relative contributions made by histology and grade to the OS and DFS difference observed across the CINSARC risk groups, we ran subgroup analyses for OS and DFS (Supplementary Tables S2 and S3). Here, the following three most relevant subgroups are reported: (i) patients with liposarcoma (WDLPS + DDLPS); (ii) all patients but those affected by SFT and WDLPS; and (iii) all patients but those affected by G1 tumors. Consistently with what was observed in the pooled cohort, in multivariable analyses, CINSARC was associated with DFS (WDLPS + DDLPS, *P* = 0.026; excluding SFT and WDLPS, *P* = 0.004; and excluding G1 tumors, *P* = 0.014), whereas it was not associated with OS in each of the three subgroups. Sarculator was significantly associated with OS in liposarcoma (WDLPS + DDLPS, *P* = 0.001) and after excluding WDLPS and SFT (*P* = 0.048). Sarculator was associated with DFS only in patients with liposarcoma (WDLPS + DDLPS, *P* = 0.013).

### Sarculator performance

Given that 16 patients in the STRASS cohort had missing data for at least one nomogram variable, Sarculator performance (with and without CINSARC) was evaluated in a total of 157 patients.

**Figure 2.**
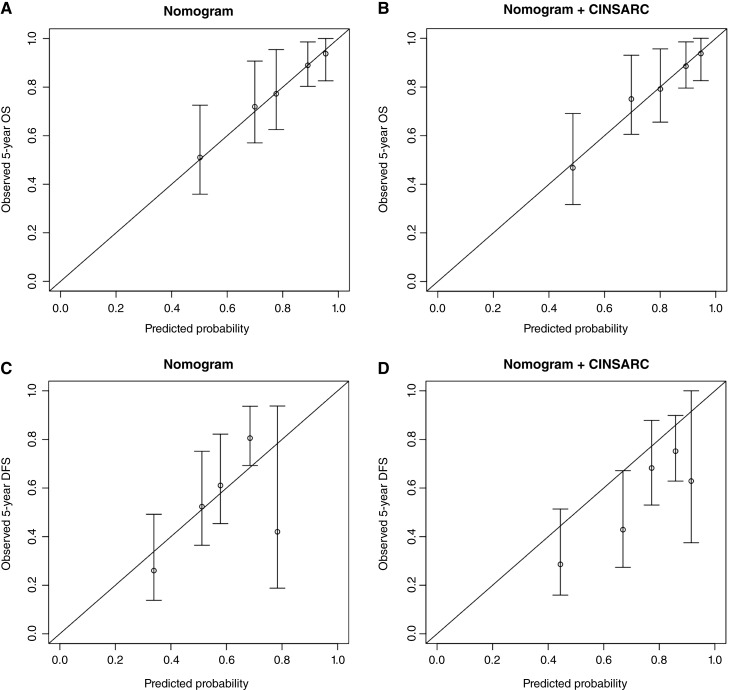
Calibration plots of the OS (**A** and **B**) and DFS (**C** and **D**) nomograms before (**A** and **C**) and after (**B** and **D**) combining CINSARC.

For OS, Sarculator’s Harrell C-index was 0.71 and remained 0.71 after incorporating CINSARC. Sarculator demonstrated good calibration for OS (Fig. 2A), and adding CINSARC did not improve calibration (Fig. 2B). The BS at 5 years was 16.4 (95% CI, 12.8–20) for Sarculator alone and 15.9 (95% CI, 12.3–19.6) after combining Sarculator and CINSARC. At 10 years, the BS was 16.5 (95% CI, 12.2–20.8) for Sarculator alone and 16.3 (95% CI, 11.6–21.0) after adding CINSARC. The bootstrapped mean delta BS at 5 years was -0.4 (95% CI, −1.1 to 0.3) and at 10 years, it was -0.2 (95% CI, −1.5 to 1.1), with neither showing statistical significance (*P* = 0.229 and *P* = 0.749, respectively).

For DFS, Sarculator’s Harrell C-index was 0.63 before combining CINSARC and 0.66 after combining CINSARC. Sarculator showed good calibration for DFS (Fig. 2C), and again, the addition of CINSARC did not improve calibration (Fig. 2D). The BS at 5 years was 22.9 (95% CI, 20.5–25.3) for Sarculator alone and 21.3 (95% CI, 18.6–24.0) after combining Sarculator and CINSARC. At 10 years, the BS was 19.9 (95% CI, 15.7–24.2) for Sarculator alone and 19.6 (95% CI, 15.0–24.2) after combining Sarculator and CINSARC. The delta BS at 5 years was −1.6 (95% CI, −3.4 to 0.1) and at 10 years it was −0.3 (95% CI, −3.9 to 3.4), with both results being nonsignificant (*P* = 0.064 and *P* = 0.884, respectively).

To assess the impact of chemotherapy and/or radiotherapy on the performance of Sarculator and CINSARC, we conducted a sensitivity analysis excluding patients who received either treatment. The predictive performance of Sarculator alone, CINSARC alone, and the combined model remained essentially unchanged (Supplementary Table S4).

**Figure 3.**
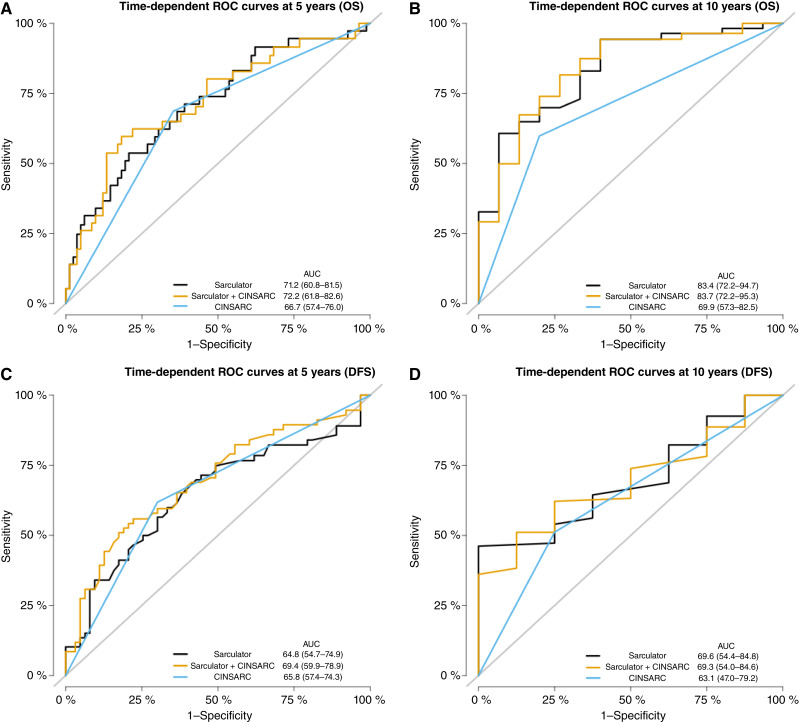
ROC curves: Time-dependent ROC curves at 5 and 10 years for OS (**A** and **B**) and DFS (**C** and **D**) in the pooled cohort, comparing different prognostic models. The Sarculator model (black), Sarculator + CINSARC (yellow), and CINSARC alone (blue) are shown. The diagonal reference line represents a nondiscriminatory model. AUC values quantify the discriminative performance of each model.

To facilitate visual comparison of the performance of the three prognostic models (Sarculator alone, CINSARC alone, and Sarculator + CINSARC), Fig. 3 presents the time-dependent ROC curves at 5 and 10 years for both OS and DFS.

In the subgroup analyses (Supplementary Tables S2 and S3), the C-index values for CINSARC were generally lower than those observed in the pooled cohort. For Sarculator, C-index values in univariable models were similar or slightly lower compared with those in the pooled cohort.

## Discussion

We validated the CINSARC signature in two distinct cohorts of patients with primary localized RPS: a retrospective cohort of 104 patients from a sarcoma referral center (INT) and a subset of 69 patients from the surgery-only arm of the EORTC-STBSG-62092 (STRASS) trial. In both cohorts, CINSARC was effective in categorizing patients into distinct groups for both OS and DFS. Specifically, there was an approximate 15% difference in OS at 4 years and an approximately 40% difference in DFS at 4 years between the CINSARC low-risk (C1) and the CINSARC high-risk (C2) groups. After pooling the two cohorts, when Sarculator and CINSARC were tested in multivariable Cox analysis, Sarculator was the only variable significantly associated with OS, whereas both Sarculator and CINSARC were associated with DFS. However, CINSARC did not improve the performance of Sarculator in terms of discrimination or calibration.

The limitations of this study include the relatively small number of patients, especially given the rarity of RPS. The combination of retrospective (INT) and prospective (STRASS) cohorts could introduce biases related to differences in patient selection or treatment protocols. Despite this limitation, all patients were treated with a standardized surgical approach in sarcoma referral centers. Furthermore, the oncologic outcomes of the STRASS and INT cohorts were comparable, and the analyses of CINSARC and Sarculator were primarily conducted on the pooled cohort. Additionally, the STRASS cohort had shorter follow-up than the INT cohort. To explore whether this difference could affect the results, we first calculated OS and DFS estimates for both cohorts—overall and stratified by CINSARC—and found no significant differences. Second, we developed a Cox mixed-effect model for OS and DFS, which showed no differences. Finally, this analysis included patients who received neoadjuvant treatment. Although we conducted sensitivity analysis to mitigate a possible impact of chemotherapy and/or radiotherapy on the performance of Sarculator and CINSARC, which did not show relevant implications, we cannot rule out a possible confounding effect for neoadjuvant therapy on prognostic effect of these two biomarkers.

This study is the first to validate CINSARC specifically in RPS. CINSARC was originally developed from a series of 183 patients with primary nontranslocated sarcoma and validated on an external independent cohort of 127 patients (14). In both the development and validation cohorts, only 21% of patients had STS located in the internal trunk, and it was not specified how many of these were RPS. In principle, a prognostic transcriptomic signature that performs well in patients with extremity and superficial trunk STS may be less effective in patients with RPS, in which the determinants of survival differ significantly.

The applications of Sarculator and CINSARC may differ in meaningful ways. Sarculator is a freely available application that leverages easily accessible variables to provide prognostic information postoperatively. This is especially relevant, as RPS characterized by intermediate tumor malignancy grade can occasionally be upgraded to high malignancy grade on final pathology (30, 31), with rare cases of multifocal disease or incomplete resection only being identified after surgery. However, it remains untested whether Sarculator would retain its predictive accuracy using solely preoperative data. In contrast, CINSARC could potentially be used preoperatively, based on tumor biopsy samples. Nonetheless, tumor heterogeneity—particularly in retroperitoneal DDLPS and LMS—may significantly impact the consistency of tumor grading between biopsy samples and final pathology (30–32). In this study, CINSARC was assessed on the surgical specimen, and there are currently no data on the correlation between CINSARC categorization on biopsy samples versus surgical specimens in RPS, nor on how tumor heterogeneity might influence CINSARC classification.

Compared with ESTS, RPS has a narrower histologic variety, with liposarcoma and LMS comprising the majority of cases (1). In RPS, patient outcomes are driven by both tumor biology and the quality of surgical resection, with local recurrence being the primary cause of death, especially in patients with liposarcoma (1, 33). This contrasts with ESTS, in which survival is predominantly driven by tumor biology, whereas the quality of resection influences local outcomes (34, 35). The Sarculator nomograms for RPS incorporate both markers of biologic aggressiveness (tumor size, FNCLCC grade, histology, and multifocality) and markers of surgical treatment quality (completeness of resection), as well as patient age (27). Given the multifaceted nature of Sarculator, which includes both biology-related (such as tumor grade and histology) and biology unrelated (such as patient’s age and completeness of resection) variables, it is unsurprising that CINSARC—a purely transcriptomic marker—alone did not enhance the prognostic performance of such a composite metric.

In the current study, high-grade tumors and LMS were more prevalent in the CINSARC high-risk (C2) group, whereas WDLPS and low-grade tumors were more commonly found in the low-risk (C1) group. This association between tumor grade and histology with CINSARC may help explain why CINSARC did not improve the performance of Sarculator as Sarculator already accounts for histologic type and tumor grade in its calculations. However, subgroup analyses—excluding WDLPS and SFT or focusing on patients with liposarcoma or grade 2 and 3 tumors—demonstrated that CINSARC remained associated with DFS in multivariable analysis. This suggests that CINSARC offers additional prognostic value beyond histology alone.

With regard to discrimination, combining CINSARC with Sarculator did not improve the Harrell C-index for either OS (remaining at approximately 0.70) or DFS (around 0.63). Calibration plots and BS similarly showed no meaningful improvement. This suggests that although CINSARC is a valuable tool for stratifying risk, it may not add further prognostic capability beyond what Sarculator already provides.

Given that CINSARC did not enhance Sarculator’s performance, further research should focus on identifying other biomarkers that may provide additional prognostic information. Our group is currently developing a transcriptomic signature with prognostic relevance for patients with RPS, which could not only be validated against Sarculator but also enhance its performance (data not shown). We have also previously identified the neutrophil-to-lymphocyte ratio as an independent prognostic factor for OS in RPS, improving Sarculator’s accuracy when added to clinical factors (13). Other potential areas for exploring new prognostic signatures include proteomics (36), radiomics (37–39), and the characterization of the tumor immune microenvironment (40, 41).

Another area worth discussing is the potential predictive role of Sarculator or CINSARC in guiding systemic therapy decisions. In the context of ESTS, several *post hoc* analyses of randomized phase III trials have consistently shown that patients with a 10-year Sarculator-predicted survival below 60% benefit from chemotherapy (42–44). However, no such threshold has been identified for patients with RPS, in which the role of chemotherapy is still under investigation in the phase III STRASS-2 trial (45, 46). This trial will provide a unique opportunity to assess whether biomarkers like Sarculator or CINSARC—which is being tested, per protocol, in all patients with available samples—could help select patients most likely to benefit from chemotherapy.

In conclusion, this study successfully validates CINSARC as a prognostic marker for OS and DFS in patients with primary RPS treated surgically. However, CINSARC did not enhance the performance of Sarculator, suggesting that its utility in improving the Sarculator nomograms may be limited. Future studies should focus on identifying additional biomarkers to further refine risk stratification and improve patient outcomes in RPS.

## Supplementary Material

Supplementary Figure S1 Supplementary Figure 1: Overall Survival curves. A, INT cohort; B, STRASS cohort; C, INT cohort according to CINSARC; D, STRASS according to CINSARC

Supplementary Figure S2 Supplemental Figure 2: Disease Free Survival curves in the INT cohort (A) and in the STRASS cohort (B) according to CINSARC

Supplementary Figure S3 Supplemental Figure 3: boxplot showing the distribution of Sarculator predicted overall survival (A, C) and Sarculator predicted disease free survival (B, D) at 5 years (A, B) and at 10 years (C, D) stratified by CINSARC risk category (blue, C1; yellow, C2). The median predicted OS/DFS is marked by the central horizontal line in each box with the box edges representing the interquartile range.

Supplementary Table S1 Supplementary Table 1: distribution of histology and grading by CINSARC for DDLPS and LMS

Supplementary Table S2 Supplementary Table 2: univariate and multivariable analyses for OS in specific subgroups

Supplementary Table S3 Supplementary Table 3: univariate and multivariable analyses for DFS in specific subgroups

Supplementary Table S4 Supplementary Table 4: sensitivity analysis excluding patients who received chemotherapy and/or radiotherapy
