# Supplementary material for: CINSARC and Sarculator in Patients with Primary Retroperitoneal Sarcoma: A Combined Analysis of Single-Institution Data and the EORTC-STBSG-62092 Trial (STRASS)
Source: Clin Cancer Res. 2025 May 27;31(15):3239–48. doi: 10.1158/1078-0432.CCR-25-0099 (PMC12314516; doi:10.1158/1078-0432.CCR-25-0099)
Supplement: Supplementary Figure S1 — Supplementary Figure 1: Overall Survival curves. A, INT cohort; B, STRASS cohort; C, INT cohort according to CINSARC; D, STRASS according to CINSARC [file ccr-25-0099_supplementary_figure_s1_suppfs1.pptx]

## Slide 1
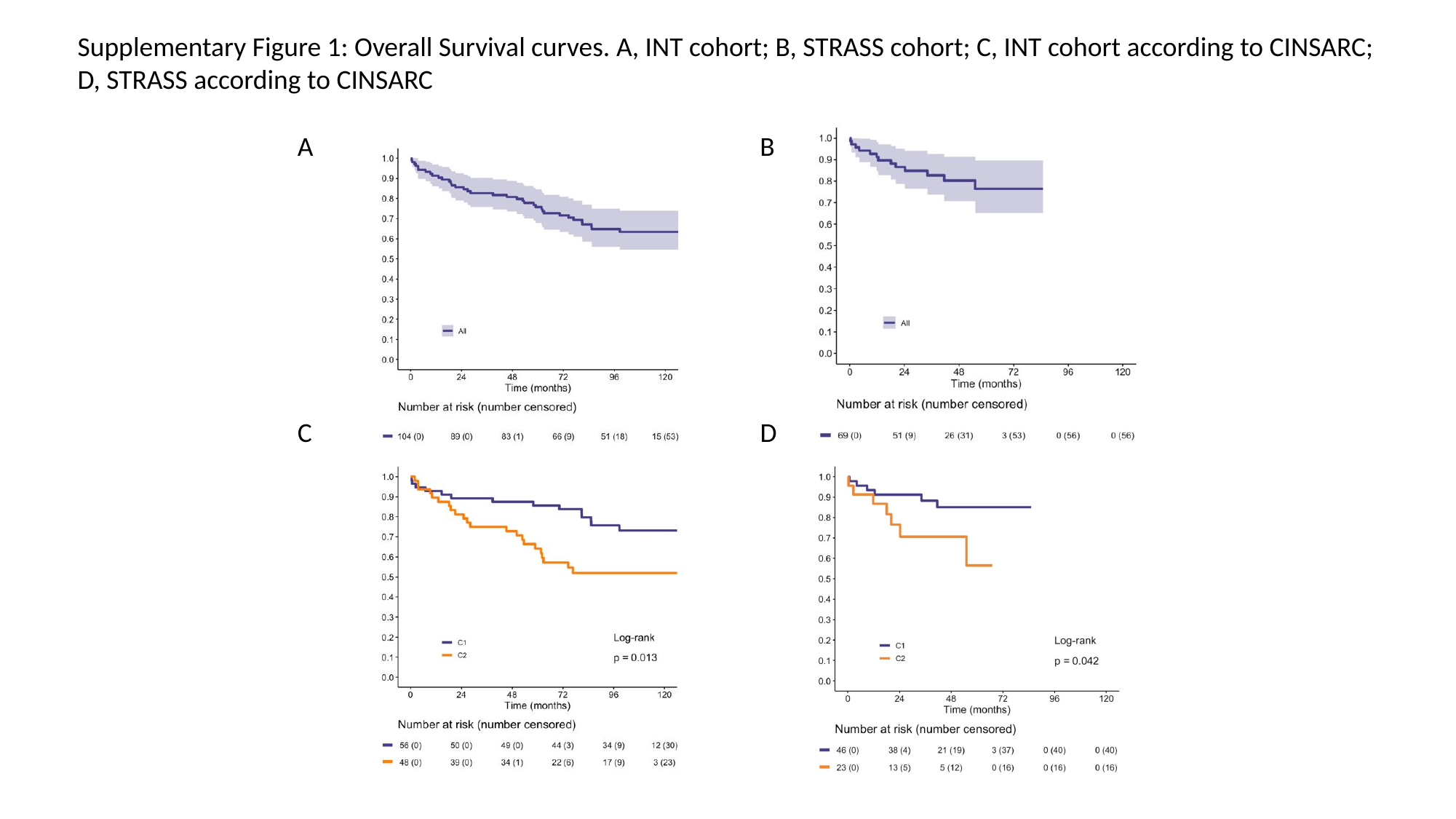

Supplementary Figure 1: Overall Survival curves. A, INT cohort; B, STRASS cohort; C, INT cohort according to CINSARC; D, STRASS according to CINSARC
A
B
C
D
