# Supplementary material for: CINSARC and Sarculator in Patients with Primary Retroperitoneal Sarcoma: A Combined Analysis of Single-Institution Data and the EORTC-STBSG-62092 Trial (STRASS)
Source: Clin Cancer Res. 2025 May 27;31(15):3239–48. doi: 10.1158/1078-0432.CCR-25-0099 (PMC12314516; doi:10.1158/1078-0432.CCR-25-0099)
Supplement: Supplementary Figure S2 — Supplemental Figure 2: Disease Free Survival curves in the INT cohort (A) and in the STRASS cohort (B) according to CINSARC [file ccr-25-0099_supplementary_figure_s2_suppfs2.pptx]

## Slide 1
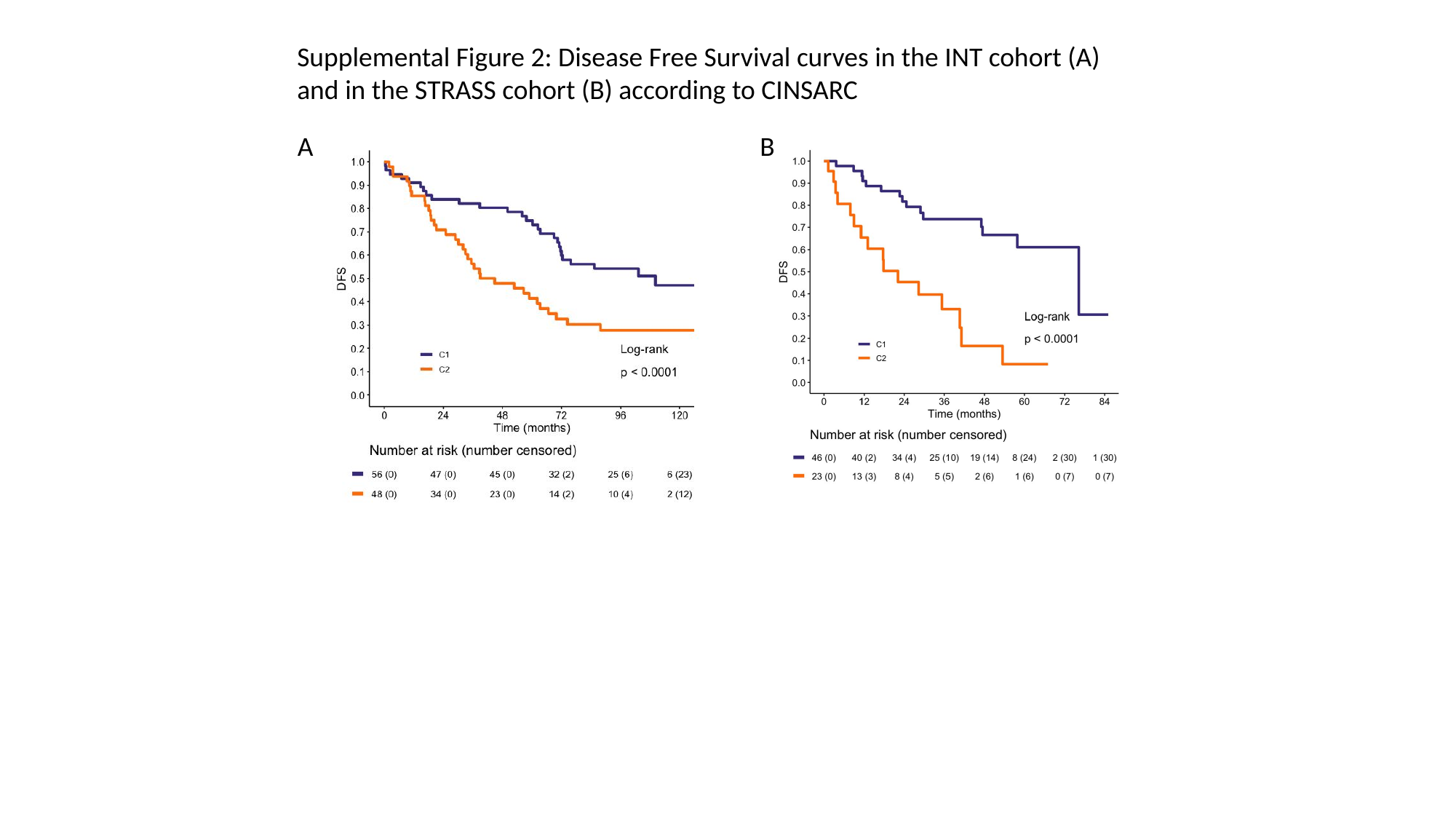

Supplemental Figure 2: Disease Free Survival curves in the INT cohort (A) and in the STRASS cohort (B) according to CINSARC
A
B
