# Supplementary material for: CINSARC and Sarculator in Patients with Primary Retroperitoneal Sarcoma: A Combined Analysis of Single-Institution Data and the EORTC-STBSG-62092 Trial (STRASS)
Source: Clin Cancer Res. 2025 May 27;31(15):3239–48. doi: 10.1158/1078-0432.CCR-25-0099 (PMC12314516; doi:10.1158/1078-0432.CCR-25-0099)
Supplement: Supplementary Figure S3 — Supplemental Figure 3: boxplot showing the distribution of Sarculator predicted overall survival (A, C) and Sarculator predicted disease free survival (B, D) at 5 years (A, B) and at 10 years (C, D) stratified by CINSARC risk category (blue, C1; yellow, C2). The median predicted OS/DFS is marked by the central horizontal line in each box with the box edges representing the interquartile range. [file ccr-25-0099_supplementary_figure_s3_suppfs3.pptx]

## Slide 1
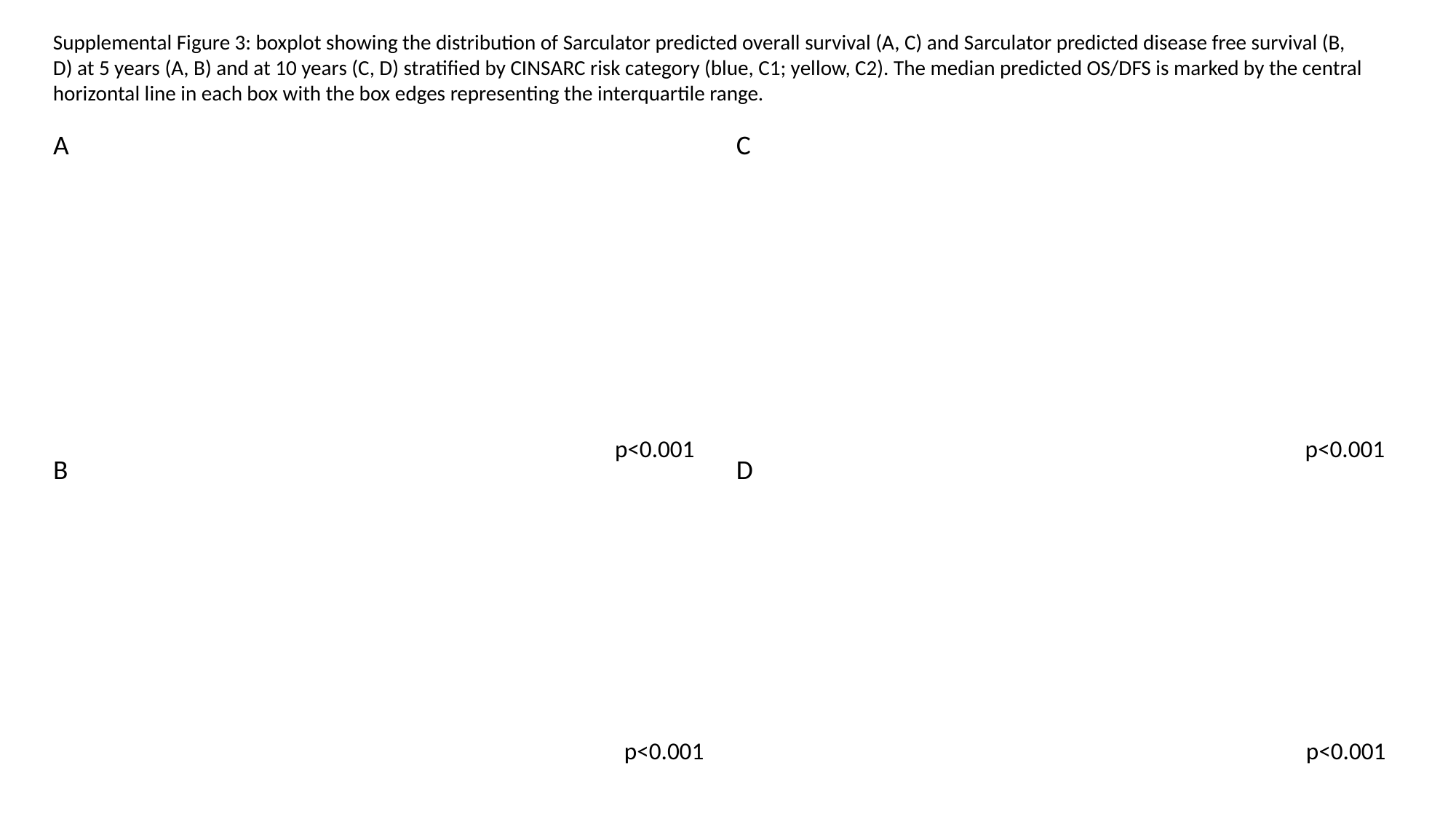

Supplemental Figure 3: boxplot showing the distribution of Sarculator predicted overall survival (A, C) and Sarculator predicted disease free survival (B, D) at 5 years (A, B) and at 10 years (C, D) stratified by CINSARC risk category (blue, C1; yellow, C2). The median predicted OS/DFS is marked by the central horizontal line in each box with the box edges representing the interquartile range.
A
C
p<0.001
p<0.001
B
D
p<0.001
p<0.001
