# Supplementary material for: CINSARC and Sarculator in Patients with Primary Retroperitoneal Sarcoma: A Combined Analysis of Single-Institution Data and the EORTC-STBSG-62092 Trial (STRASS)
Source: Clin Cancer Res. 2025 May 27;31(15):3239–48. doi: 10.1158/1078-0432.CCR-25-0099 (PMC12314516; doi:10.1158/1078-0432.CCR-25-0099)
Supplement: Supplementary Table S1 — Supplementary Table 1: distribution of histology and grading by CINSARC for DDLPS and LMS [file ccr-25-0099_supplementary_table_s1_suppts1.docx]

Supplementary Table 1: distribution of histology and grading by CINSARC for DDLPS and LMS

| **Characteristic** | **C1 (N=102)** | **C2 (N=71)** | **p** |
| --- | --- | --- | --- |
| **DDLPS (N C1=47; N C2=32)** | | | 0.003 |
| G1 DDLPS | 3 (7.5%) | 1 (3.2%) |  |
| G2 DDLPS | 32 (80%) | 15 (48.4%) |  |
| G3 DDLPS | 5 (12.5%) | 15 (48.4%) |  |
| G Unknown | 7 | 1 |  |
| **LMS - Grading (N C1=5; N C2=22)** | | | 0.110 |
| G1 LMS | 2 (50%) | 3 (14.2%) |  |
| G2 LMS | 2 (50%) | 9 (42.8%) |  |
| G3 LMS | 0 | 9 (42.8%) |  |
| G Unknown | 1 | 1 |  |
