# Supplementary material for: CINSARC and Sarculator in Patients with Primary Retroperitoneal Sarcoma: A Combined Analysis of Single-Institution Data and the EORTC-STBSG-62092 Trial (STRASS)
Source: Clin Cancer Res. 2025 May 27;31(15):3239–48. doi: 10.1158/1078-0432.CCR-25-0099 (PMC12314516; doi:10.1158/1078-0432.CCR-25-0099)
Supplement: Supplementary Table S2 — Supplementary Table 2: univariate and multivariable analyses for OS in specific subgroups [file ccr-25-0099_supplementary_table_s2_suppts2.docx]

Supplementary Table 2: univariate and multivariable analyses for OS in specific subgroups

| **Subgroup** | **OS – univariabe Cox models** | | | | **OS – multivariable Cox models** | | | |
| --- | --- | --- | --- | --- | --- | --- | --- | --- |
|  | **HR** | **95% CI** | **p** | **C-index** | **HR** | **95% CI** | **p** | **C-index** |
| **DDLPS** | | | | | | | | |
| Sarculator | 1.69 | (0.95 - 2.99) | 0.0740 | 0.616 | 1.44 | (0.77 - 2.7) | 0.2500 | 0.610 |
| CINSARC | 1.59 | (0.78 - 3.26) | 0.2000 | 0.541 | 1.53 | (0.64 - 3.67) | 0.3300 |  |
| **WDLPS** | | | | | | | | |
| Sarculator | 85.25 | (5.62 - 1293.23) | 0.0014 | 0.770 | 94.23 | (5.7 - 1558.54) | 0.0015 | 0.774 |
| CINSARC | 1.41 | (0.27 - 7.29) | 0.6800 | 0.518 | 1.72 | (0.32 - 9.33) | 0.5300 |  |
| **WDLPS + DDLPS** | | | | | | | | |
| Sarculator | 2.13 | (1.48 - 3.06) | 0.0000 | 0.714 | 1.98 | (1.32 - 2.97) | 0.0009 | 0.708 |
| CINSARC | 1.95 | (1.02 - 3.73) | 0.0430 | 0.568 | 1.33 | (0.62 - 2.86) | 0.4700 |  |
| **LMS** | | | | | | | | |
| Sarculator | 1.75 | (0.73 - 4.17) | 0.2100 | 0.706 | 1.54 | (0.64 - 3.69) | 0.3300 | 0.742 |
| CINSARC | 2.60e+8 | (0 - Inf) | 1.0000 | 0.607 | 1.65e+8 | (0 - Inf) | 1.0000 |  |
| **LMS + DDLPS +UPS + MPNST + Other** | | | | | | | | |
| Sarculator | 1.83 | (1.16 - 2.89) | 0.0093 | 0.643 | 1.59 | (1.00 - 2.53) | 0.0480 | 0.653 |
| CINSARC | 1.86 | (0.99 - 3.5) | 0.0540 | 0.577 | 1.72 | (0.83 - 3.56) | 0.1500 |  |
| **Grade I** | | | | | | | | |
| Sarculator | 5.47 | (0.66 - 45.66) | 0.1200 | 0.682 | 5.75 | (0.69 - 47.71) | 0.1100 | 0.663 |
| CINSARC | 2.05 | (0.49 - 8.59) | 0.3300 | 0.568 | 2.23 | (0.53 - 9.39) | 0.2800 |  |
| **Grade II - Grade III** | | | | | | | | |
| Sarculator | 2.10 | (1.22 - 3.59) | 0.0072 | 0.628 | 1.77 | (0.98 - 3.20) | 0.0580 | 0.641 |
| CINSARC | 2.12 | (1.07 - 4.21) | 0.0320 | 0.595 | 1.60 | (0.75 - 3.42) | 0.2300 |  |
