# Supplementary material for: CINSARC and Sarculator in Patients with Primary Retroperitoneal Sarcoma: A Combined Analysis of Single-Institution Data and the EORTC-STBSG-62092 Trial (STRASS)
Source: Clin Cancer Res. 2025 May 27;31(15):3239–48. doi: 10.1158/1078-0432.CCR-25-0099 (PMC12314516; doi:10.1158/1078-0432.CCR-25-0099)
Supplement: Supplementary Table S3 — Supplementary Table 3: univariate and multivariable analyses for DFS in specific subgroups [file ccr-25-0099_supplementary_table_s3_suppts3.docx]

Supplementary Table 3: univariate and multivariable analyses for DFS in specific subgroups

| **Subgroup** | **DFS – univariabe Cox models** | | | | **DFS – multivariable Cox models** | | | |
| --- | --- | --- | --- | --- | --- | --- | --- | --- |
|  | **HR** | **95% CI** | **p** | **C-index** | **HR** | **95% CI** | **p** | **C-index** |
| **DDLPS** | | | | | | | | |
| Sarculator | 1.95 | (1.08 - 3.50) | 0.0260 | 0.581 | 1.36 | (0.72 - 2.57) | 0.3400 | 0.614 |
| CINSARC | 2.15 | (1.18 - 3.89) | 0.0120 | 0.572 | 2.24 | (1.09 - 4.60) | 0.0270 |  |
| **WDLPS** | | | | | | | | |
| Sarculator | 0.18 | (0.01 - 4.10) | 0.2800 | 0.535 | 0.17 | (0.01 - 4.11) | 0.2800 | 0.524 |
| CINSARC | 0.97 | (0.36 - 2.62) | 0.9600 | 0.503 | 0.92 | (0.33 - 2.54) | 0.8700 |  |
| **WDLPS + DDLPS** | | | | | | | | |
| Sarculator | 1.89 | (1.32 - 2.72) | 0.0006 | 0.650 | 1.61 | (1.11 - 2.36) | 0.0130 | 0.659 |
| CINSARC | 1.98 | (1.22 - 3.22) | 0.0054 | 0.581 | 1.84 | (1.08 - 3.15) | 0.0260 |  |
| **LMS** | | | | | | | | |
| Sarculator | 1.12 | (0.58 - 2.18) | 0.7300 | 0.563 | 0.92 | (0.46 - 1.85) | 0.8200 | 0.555 |
| CINSARC | 4.96 | (0.66 - 37.43) | 0.1200 | 0.594 | 4.40 | (0.55 - 35.3) | 0.1600 |  |
| **LMS + DDLPS +UPS + MPNST + Other** | | | | | | | | |
| Sarculator | 1.50 | (0.98 - 2.30) | 0.0630 | 0.554 | 1.17 | (0.76 - 1.80) | 0.4800 | 0.609 |
| CINSARC | 2.27 | (1.38 - 3.73) | 0.0013 | 0.594 | 2.31 | (1.31 - 4.08) | 0.0039 |  |
| **Grade I** | | | | | | | | |
| Sarculator | 1.11 | (0.39 - 3.15) | 0.8500 | 0.434 | 1.06 | (0.36 - 3.10) | 0.9100 | 0.555 |
| CINSARC | 1.61 | (0.70 - 3.70) | 0.2600 | 0.560 | 1.60 | (0.69 - 3.70) | 0.2700 |  |
| **Grade II - Grade III** | | | | | | | | |
| Sarculator | 1.82 | (1.05 - 3.16) | 0.0340 | 0.553 | 1.30 | (0.7 - 2.38) | 0.4100 | 0.598 |
| CINSARC | 2.35 | (1.36 - 4.09) | 0.0023 | 0.593 | 2.13 | (1.16 - 3.89) | 0.0140 |  |
