# Supplementary material for: CINSARC and Sarculator in Patients with Primary Retroperitoneal Sarcoma: A Combined Analysis of Single-Institution Data and the EORTC-STBSG-62092 Trial (STRASS)
Source: Clin Cancer Res. 2025 May 27;31(15):3239–48. doi: 10.1158/1078-0432.CCR-25-0099 (PMC12314516; doi:10.1158/1078-0432.CCR-25-0099)
Supplement: Supplementary Table S4 — Supplementary Table 4: sensitivity analysis excluding patients who received chemotherapy and/or radiotherapy [file ccr-25-0099_supplementary_table_s4_suppts4.docx]

Supplementary Table 4: sensitivity analysis excluding patients who received chemotherapy and/or radiotherapy

| **Sensitivity Analysis**  **(No RT/CT Neoadj-Adj. Subgroup)** | **OS** | | | | **DFS** | | | |
| --- | --- | --- | --- | --- | --- | --- | --- | --- |
|  | **HR** | **95% CI** | **p** | **C-index** | **HR** | **95% CI** | **p** | **C-index** |
| **Univariabe Cox models** | | | | | | | | |
| Sarculator | 2.18 | (1.55 - 3.06) | <0.001 | 0.719 | 1.98 | (1.42 - 2.78) | <0.001 | 0.620 |
| CINSARC | 2.62 | (1.40 - 4.92) | 0.0030 | 0.615 | 2.38 | (1.53 - 3.71) | <0.001 | 0.635 |
| **Multivariable Cox models** | | | | | | | | |
| Sarculator | 1.88 | (1.29 - 2.73) | <0.001 | 0.716 | 1.61 | (1.12 - 2.30) | 0.0100 | 0.671 |
| CINSARC | 1.78 | (0.83 - 3.82) | 0.1400 |  | 1.97 | (1.18 - 3.30) | 0.0100 |  |
